# Dietary Nutrients and Cardiovascular Risk Factors among Renal Transplant Recipients

**DOI:** 10.3390/ijerph18168448

**Published:** 2021-08-10

**Authors:** I-Hsin Lin, Tuyen Van Duong, Te-Chih Wong, Shih-Wei Nien, I-Hsin Tseng, Yang-Jen Chiang, Hsu-Han Wang, Shwu-Huey Yang

**Affiliations:** 1 Department of Medical Nutrition Therapy, Linkou Chang Gung Memorial Hospital, Linkou 33305, Taiwan; cabbage@cgmh.org.tw (I.-H.L.); nina0904@cgmh.org.tw (S.-W.N.); cathy40422@cgmh.org.tw (I.-H.T.); 2 School of Nutrition and Health Sciences, College of Nutrition, Taipei Medical University, Taipei 11031, Taiwan; duongtuyenvna@gmail.com; 3 Department of Nutrition and Health Sciences, Chinese Culture University, Taipei 11114, Taiwan; Wdz5@ulive.pccu.edu.tw; 4 Department of Urology, Linkou Chang Gung Memorial Hospital, Linkou 33305, Taiwan; zorro@cgmh.org.tw (Y.-J.C.); seanwang@cgmh.org.tw (H.-H.W.); 5 Research Center of Geriatric Nutrition, College of Nutrition, Taipei Medical University, Taipei 11031, Taiwan; 6 Nutrition Research Center, Taipei Medical University Hospital, Taipei 11031, Taiwan

**Keywords:** dietary nutrient intake, kidney transplant, renal transplant recipients, cardiovascular disease, cardiovascular disease risk factor

## Abstract

Cardiovascular disease (CVD) is the leading cause of mortality in post-renal transplant recipients (RTRs). Adequate nutrient intake is a protective factor for CVD. We examined the associations of macronutrients and micronutrients with traditional and nontraditional CVD risk factors. Conducted from September 2016 to June 2018, this cross-sectional study included 106 RTRs aged ≥18 years with a functioning allograft. Dietary intake data from 3-day dietary records were collected. Nutrient intake adequacy was defined using various instruments, including the National Kidney Foundation Kidney Disease Outcomes Quality Initiative (K/DOQI) guidelines. CVD risk factors were defined according to the K/DOQI guidelines. Bivariate and multivariate logistic regression models were used to analyze the associations. CVD risk was present in all patients; the lowest proportions of adequate intake were 2.8% for dietary fiber and 0.9% for calcium. Adequate nutrient intake was associated with a lower likelihood of the occurrence of traditional CVD risk factors (specifically, 1.9–31.3% for hyperlipidemia and 94.6% for diabetes mellitus). It was also associated with a lower likelihood of the occurrence of nontraditional CVD risk by 0.8% for hypophosphatemia and 34% for hyperuricemia. Adherence to dietary guidelines should be promoted among RTRs to decrease CVD risk.

## 1. Introduction

Among renal replacement therapies, renal transplant therapy (RTT) provides renal transplant recipients (RTRs) with the best quality of life [1]. RTT has a higher 5-year survival rate (89.6%) than end-stage renal disease (58.7%) and dialysis (57.3%) [2]. However, CVD remains the leading cause of mortality after RTT and accounts for 100% of deaths from chronic kidney disease in Taiwan [3].

CVD risk factors are outlined by the National Kidney Foundation according to the Kidney Disease Outcomes Quality Initiative (K/DOQI) [4,5,6,7] Clinical Practice Guidelines for CVD in chronic kidney disease. Traditional factors (e.g., older age, male gender, high blood pressure, high plasma glucose levels, abnormal lipid profile, and obesity) and nontraditional CVD risk factors (e.g., anemia, chronic inflammation, and electrolyte and mineral metabolism abnormalities) [8,9] alike are strongly associated with high CVD prevalence and mortality [10,11].

Various intervention strategies, such as medication, nutrition, and lifestyle modification, are needed for early CVD prevention [12]. Nutritional interventions, including better nutritional status and adherence to individual dietary recommendations, lead to lower metabolic risk and longer kidney survival rate, respectively [13,14]. However, most RTRs show improved appetite after the lifting of dietary restrictions and do not meet the dietary requirements for CVD risk reduction [15,16,17]. Poor adherence to dietary strategies can contribute to increasing morbidity and mortality, as shown in a previous study [18].

The role of dietary nutrient intake in CVD risk factors for RTRs remains to be examined. Therefore, we investigated the associations of adequate dietary macronutrient and micronutrient intake with traditional and nontraditional CVD risk factors in stable RTRs at a single medical center in Taiwan. We hypothesized that RTRs with adequate nutrient intake would have a lower likelihood of the occurrence of CVD risk factors.

## 2. Material and Methods

### 2.1. Study Design and Participants

**Figure 1:**
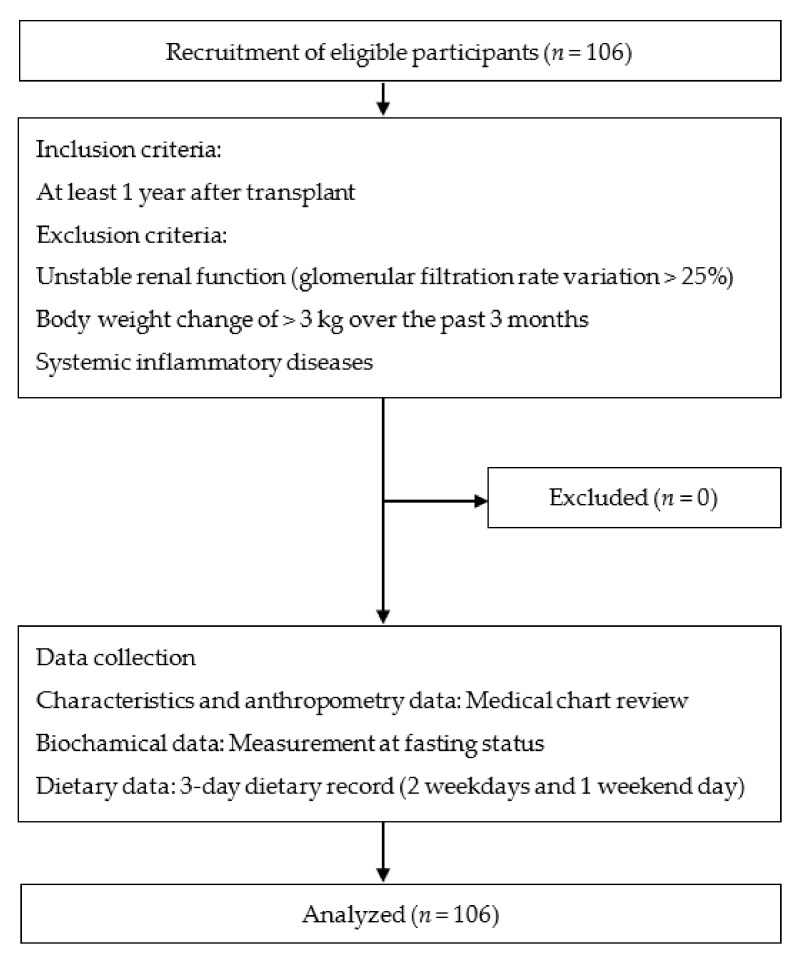
Study procedure and flowchart with patients’ enrollment.

A total of 106 RTRs were enrolled in this cross-sectional study, which was conducted between September 2016 and June 2018 at Linkou Chang Gung Memorial Hospital, Taoyuan, Taiwan. We included patients aged ≥18 years who had had a functioning allograft for at least 1 year after transplant. Patients with unstable renal function, glomerular filtration rate variation >25%, body weight change of >3 kg over the past 3 months, and systemic inflammatory diseases were excluded [17]. The study sample was illustrated in Figure 1.

A registered dietitian at the participating hospital interviewed the participants in person. Data such as anthropometry, laboratory, and dietary intake were also collected according to standardized methods. Informed consent was obtained from each RTR before the interview. The study protocol was approved by the Chang Gung Medical Foundation’s Institutional Review Board (No. 201600954B0).

### 2.2. Patient Characteristics and Laboratory Parameters

Patient characteristics, which were obtained using medical records, included age, gender, dialysis history, transplant history, body height, body weight, and blood pressure. Body mass index (BMI) was calculated as weight in kilograms divided by the square of height in meters. Waist circumference (WC) in centimeters was noted, and body composition was assessed on Omron scales (HBF-375; Omron Healthcare, Kyoto, Japan) through bioelectrical impedance analysis. Patients fasted for >8 h before examination and wore indoor clothing.

The following parameters were analyzed with standardized methods, as described previously [17]: estimated glomerular filtrate rate (eGFR), fasting plasma glucose, total cholesterol (TC), triglycerides (TG), low-density lipoprotein cholesterol (LDL-C), high-density lipoprotein cholesterol (HDL-C), uric acid, albumin, high-sensitivity C-reactive protein (hs-CRP), serum calcium, phosphate, potassium, hemoglobin (Hb), and intact parathyroid hormone (iPTH). In the homeo-static model assessment of insulin resistance (HOMA-IR), the following formula was used [19]: HOMA-IR = fasting insulin (µU/mL) × fasting glucose (mmol/L)/22.5.

The characteristics and CVD risk factors of the participants are presented in Table 1.

### 2.3. Traditional and Nontraditional CVD Risk Factors

The traditional risk factors of CVD are older age (>65 years); male gender [20,21]; obesity, defined as BMI ≥ 24 kg/m^2^ [25] on the basis of the Taiwan Ministry of Health and Welfare (MOHW) guidelines; high WC, defined as ≥90 cm for men and ≥80 cm for women [26]; and total body fat (TBF) ≥ 25% and ≥30% for men and women, respectively [22]. Other risk factors include hypertension, diabetes mellitus, and dyslipidemia. Hypertension [27] was defined as systolic blood pressure ≥130 mmHg and diastolic blood pressure ≥80 mmHg. Diabetes mellitus [28] was defined as fasting plasma glucose ≥126 mg/dL and examined with regard to median HOMA-IR values ≥ 1.44. Dyslipidemia was defined according to the third report of the National Cholesterol Education Program in Adult Treatment Panel III (NCEP-ATP III) [29]: serum TC ≥ 200 mg/dL, serum TG ≥ 150 mg/dL, and serum LDL-C ≥ 100 mg/dL. The cutoff for serum HDL-C is under 40 mg/dL for men and under 50 mg/dL for women.

Among nontraditional CVD risk factors, anemia [6] was defined as Hb concentrations <12 g/dL for men and <13 g/dL for women. Poor renal function, chronic kidney disease stage 3b–5, was determined by an eGFR of <45 according to the K/DOQI guidelines. A concentration exceeding 0.5 mg/dL for hs-CRP, an inflammatory marker [23], was considered a risk factor for CVD. Hyperuricemia [30] was defined as uric acid concentration ≥7.0 mg/dL. Albumin-corrected calcium was measured as total calcium (mg/dL) + 0.8 × (4.0−serum albumin [in grams per deciliter]) [31]. The cutoffs for abnormal serum calcium, serum phosphorus, and serum potassium were <8.4 mg/dL, <3.5 mg/dL [5], and ≥5.0 mg/dL [31], respectively. Finally, the cutoff for intact parathyroid hormone (iPTH) was ≥150 pg/mL [6].

### 2.4. Dietary Intake

The participants completed dietary records over 3 days (2 weekdays and 1 weekend day) before visiting the dietitian for the latest follow-up session. In order to confirm dietary records, 24 h dietary recall was completed through face-to-face interviews by a well-trained dietitian. Dietary energy and nutrient intake were calculated using nutrition analysis software (Cofit Pro version 1.0.0, Cofit HealthCare Inc., Taipei, Taiwan) [32]. Macronutrient and micronutrient intakes were calculated according to guidelines presented in the database of the Taiwan MOHW [33]. The following guidelines were used for renal disease: the guidelines for the nutritional management of adult kidney transplant recipients [25], K/DOQI guidelines [4,5,6,7], the European Renal Best Practice Guidelines [34], and the MOHW dietary reference intakes [35]. The macronutrients and micronutrients examined and their intakes are presented by category in Table 2.

### 2.5. Statistical Analysis

Analyses were performed using SAS software, Version 9.4 of the SAS System (SAS Institute Inc., Cary, NC, USA). Data are presented as means ± standard deviations, medians (interquartile ranges), or percentages. To comprehensively evaluate the association between nutrient intake adequacy and CVD risk factors, multivariate logistic regression was conducted (with adjustments for age, gender, renal function, trans-plant vintage, and the Charlson comorbidity index) to estimate the odds ratios (ORs) and 95% confidence intervals (95% CIs). The significance level was set at *p* < 0.05.

## 3. Results

### 3.1. Characteristics and CVD Risk Factors of RTRs

A total of 106 eligible RTRs were analyzed. The means of patient age, duration of dialysis treatment, and transplant vintage were 48.9 ± 12.8, 6.5 ± 4.9, and 8.6 ± 5.9 years, respectively. CVD risk factors were as follows: around 7.5% of the patients were older adults and 57.5% were men; regarding obesity, 41.5% had high WC, 51.9% had high BMI, and 50.9% had high TBF and abnormal plasma glucose and lipid profiles, 88.7% had high fasting plasma glucose, 50.1% had high TC, 65.1% had high LDL-C, 38.7% had low HDL-C, and 30.2% had high TG. All RTRs had CVD risk factors ranging from 4 to 14 risks (Table 1).

### 3.2. The Proportion of RTRs with Adequate Nutrient Intake

Overall, 27.4%, 18.0%, 20.8%, and 21.7% of the patients had adequate intakes of energy, protein, carbohydrates, and fat, respectively. Less than half of the RTRs reached the recommendations of nutrient intake, except for dietary MUFA (95.3%), vitamin A (50.0%), and C (59.4%), which were noted (Table 2).

### 3.3. Nutrient Intake Adequacy and Traditional CVD Risk Factors

The proportions of nutrients by traditional CVD risk factors are presented in Table S1. Adequate energy intake was significantly associated with lower odds of high median of HOMA-IR (OR, 0.05; 95% CI, 0.01–0.81, *p* = 0.003). Adequate vitamin A intake was significantly associated with lower odds of high TC (OR, 0.69; 95% CI, 0.52–0.91, *p* = 0.009). Adequate vitamin E intake was significantly associated with lower odds of low HDL-C (OR, 0.98; 95% CI, 0.96–1.00, *p* = 0.04). An unexpected result was the significant association of adequate carbohydrate intake with higher odds of high TG (OR, 1.26; 95% CI, 1.01–1.57, *p* = 0.04) (Table 3).

### 3.4. Nutrient Intake Adequacy and Nontraditional CVD Risk Factors

The proportions of nutrients by non-traditional CVD risk factors are presented in Table S2. Adequate intake of polyunsaturated fatty acids was significantly associated with lower odds of having low serum phosphate (OR, 0.99; 95% CI, 0.99–1.00, *p* = 0.04). Adequate phosphate intake was significantly associated with lower odds of hyperuricemia (OR, 0.66; 95% CI, 0.46–0.94, *p* = 0.02). Unexpected results were the significant association of adequate vitamin B_2_ intake (OR, 1.23; 95% CI, 1.01–1.50, *p* = 0.046) and iron intake (OR, 1.34; 95% CI, 1.01–1.80, *p* = 0.048) with high odds of anemia, as well as the significant association of adequate protein intake with high odds of hyperuricemia (OR, 1.32; 95% CI, 1.03–1.70, *p* = 0.03) (Table 4).

## 4. Discussion

Only a low proportion of the participants had adequate macronutrient and micronutrient intakes, consistent with a previous observation of poor compliance with dietary guidelines in stable RTRs [17]. Adequate nutrient intake was significantly associated with lower likelihoods of the occurrence of multiple traditional and nontraditional CVD risk factors.

A lower proportion of RTRs in the present study had adequate energy intake compared with that in a previous study [36] (27.4% vs. 51.6%) in which 31 Japanese renal allograft recipients were followed for more than 10 years. However, the proportions of patients with adequate protein intake (87.4% vs. 87%) were comparable. A higher proportion of the present participants had adequate fat intake (39.8% vs. 29%) and high median carbohydrate intake (205.9 vs. 196 g). Notably, the present study had higher proportions of RTRs with high TC (50.1%) and high LDL-C (65.1%) than did a study on Taiwanese patients receiving hemodialysis treatment (18.7% and 48.4%, respectively). By contrast, the proportions of RTRs with low HDL-C and TG (38.7% and 30.2%, respectively) were lower than those in a previous study (65.9% and 42.7%, respectively) [37]. These comparisons indicate that patients’ lipid profiles may remain abnormal even after they receive renal transplants.

CVD is a major cause of mortality in RTRs [15]. Adequate intakes of energy, protein, and carbohydrates can reduce CVD risk in patients with chronic kidney disease [4,5,6,7,25,36]. No significant difference was noted in the present study between adequate protein and carbohydrate intakes and reductions in CVD risk, but adequate carbohydrate intake was associated with higher odds of high TG. Immunosuppression medication, such as a mammalian target of rapamycin (mTOR) inhibitor, among RTRs may affect serum lipid metabolism [38]. In the present study, a total of 61 RTRs (57.5%) were receiving mTOR medication and 12 RTRs (19.6%) had high serum TG (data were not shown in table.). In addition, the association of high serum TG with the high intake of simple sugars in RTRs, especially fructose, is attributable to high endotoxin concentrations [39], which reduces satiety and increases appetite [40] through the impaired or failed induction of the postprandial leptin response or the inhibition of ghrelin suppression. Further studies on various sugar-containing foods are warranted to examine the effect of carbohydrate or simple sugar intake on CVD risk factors.

Adequate protein intake was associated with high odds of hyperuricemia in the present study, in line with results from a cross-sectional study including 3978 middle-aged men—specifically, a positive association between hyperuricemia and high intake of animal protein (e.g., red meat and seafood) and a negative association between hyperuricemia with the high intake of purine-rich vegetables and soy-containing foods [41]. In the present study, 82% of RTRs had adequate or high protein intake (1.12 g ± 0.3 g/kg), suggesting that the association between hyperuricemia and different protein sources should be further examined to facilitate guidance in nutritional therapy.

Regarding fat intake, no significant difference was observed between fatty acid intake and serum TG, TC, LDL-C, and HDL-C, consistent with results from a study on patients receiving hemodialysis treatment [39,42]. However, adequate polyunsaturated fatty acid intake was associated with lower odds of hypophosphatemia in the present study, supporting the argument that adequate intake of the class of fatty acids is essential for RTRs [7]. Further research is required to evaluate the association between n-3 polyunsaturated fatty acids and CVD risk factors.

Adequate intakes of vitamins A and E were significantly associated with lower odds of hypercholesterolemia and low HDL-C, in line with the assertion that the abundance of dietary antioxidants in vitamins A, C, E, and D helps to prevent CVD [43]. Moreover, deficiencies in vitamins A, C, E, and D are correlated with high risk of metabolic syndrome [44]. This may be ascribable to the fact that adequate vitamin intake is associated with lower mean DNA methylation levels of ATP-binding cassette transporter A1, which is in turn inversely associated with HDL-C metabolism [45].

Adequate phosphate intake was associated with lower odds of hyperuricemia, a result supported by the inverse association between serum phosphate levels and hyperuricemia reported by Cao et al. [46]. This may be because low serum phosphate levels strongly induce DNA damage [47] and contribute to the release of purine nucleotides; the degradation of AMP to inosine monophosphate by adenosine deaminase in hypophosphatemia may accelerate the synthesis of uric acid [48]. Notably, a previous study also elucidated a link between dietary and serum phosphorus concentrations and CVD events in patients receiving hemodialysis treatment [49].

Our study has several strengths and limitations. First, to the best of our knowledge, this is the first study to investigate the associations between nutrient intake and CVD risk in RTRs. However, causality cannot be inferred from the results because of its cross-sectional nature; further well-designed prospective studies and randomized controlled trials are warranted to determine whether the present findings are generalizable to other populations of RTRs. Second, the contribution of diet to metabolic risk in RTRs may be dependent on food composition. Further studies should be conducted with subgroups of the various types of dietary carbohydrates, proteins, and fatty acids. Third, although this was not a randomized controlled trial, our results were analyzed using reliable laboratory data, epidemiological methods, and various comprehensive tools and measures (e.g., 3-day dietary records with 24 h recall) to determine dietary quality. These methods have been used to assess the dietary intake of RTRs, examine their nutrition-related problems [32,37,50], and raise awareness about their nutritional status and CVD risk. Finally, the results are not generalizable to other areas of world where the diet may be different, and the small sample size precluded the examination of the interactions between nutrients. For example, dietary calcium, phosphate, and magnesium can interact with fatty acids; they reduce the absorption of saturated fatty acids and are positively correlated with high serum TC, among other CVD risk factors [51].

## 5. Conclusions

The present study comprehensively analyzed the dietary intake of macronutrients and micronutrients and their associations with traditional and nontraditional CVD risk factors among RTRs. Adequate nutrient intake was associated with a 94.6% lower risk of CVD. Dietary recommendations for RTRs should emphasize adherence to dietary guidelines for CVD prevention. Further longitudinal studies and randomized controlled trials are warranted to verify our findings.

## Figures and Tables

**Table 1:** Characteristics and cardiovascular risk factors in renal transplant recipients (*n* = 106).

Variables	All Patients Mean ± SD		Patients with CVD Risk Factors *n* (%)		CVD Risk Factors
Characteristics					
Age, y	48.9	12.8	8	(7.5)	<65 ^1,2^
Gender			61	(57.5)	Men ^1,2^
Hemodialysis vintage, y	6.5	4.9			
Transplant vintage, y	8.6	5.9			
Hypertension			94	(88.6)	
Dyslipidemia			34	(32.1)	
Diabetes mellitus			23	(21.7)	
Traditional CVD risk factors					
WC, cm	83.4	9.8	44	(41.5)	≥90 for male; ≥80 for female ^3^
BMI, kg/m^2^	24.0	3.7	55	(51.9)	≥ 24 ^3^
TBF, %	26.3	7.4	54	(50.9)	≥25 for male; ≥30 for female ^4^
SBP, mm Hg	132.6	15.9	63	(59.4)	≥130 ^5^
DBP, mm Hg	77.5	11.8	42	(39.6)	≥80 ^5^
FPG, mg/dL	126.9	24.0	94	(88.7)	≥126 ^6^
HOMA-IR	2.3	4.5	53	(50.0)	≥1.44 (median)
TC, mg/dL	205.4	43.6	54	(50.1)	≥200 ^7^
LDL-C, mg/dL	118.9	38.0	69	(65.1)	≥100 ^7^
HDL-C, mg/dL	52.0	17.7	41	(38.7)	<40 for male; <50 for female ^7^
TG, mg/dL	156.1	118.0	32	(30.2)	≥150 ^7^
Nontraditional CVD risk factors					
Hemoglobin, g/dL	12.6	3.8	51	(48.1)	<12 for male; <13 for female ^8^
Serum creatinine, mg/dL	1.49	0.89	52	(49.1)	≥1.29 (median)
eGFR, ml/min/1.73 m^2^	55.1	20.7	33	(31.1)	≤45; CKD stage 3b–5 ^8^
Corrected calcium, mg/dL	9.2	0.9	11	(10.4)	<8.4 ^8^
Serum phosphate, mg/dL	3.5	1.0	57	(53.8)	<3.5 ^8^
iPTH, pg/mL	116.1	123.7	18	(17.0)	≥150 ^8^
hs-CRP, mg/dL	4.43	5.86	87	(82.1)	>0.5 ^9^
Uric acid, g/dL	6.3	1.3	36	(34.0)	<7.0 ^10^
Serum potassium, mg/dL	4.4	0.5	12	(11.4)	≥5.0 ^11^
Numbers of CVR risk factors	8.3	2.7			

Y, year; CVD, cardiovascular disease; WC, waist circumference; BMI, body mass index; TBF, total body fat; SBP, systolic blood pressure; DBP, diastolic blood pressure; FPG, fasting plasma glucose; TC, total cholesterol; LDL-C, low-density lipoprotein cholesterol; HDL-C, high-density lipoprotein cholesterol; TGs, triglycerides; eGFR, estimated glomerular filtration rate; CKD, chronic kidney disease; hs-CRP, high-sensitivity C-reactive protein; iPTH, intact parathyroid hormone. ^1^ The diagnostic values were defined by Sarnak and Levey [20] ^2^ The diagnostic values were defined by Yerram et al. [21]. ^3^ The diagnostic values were defined by the Taiwan Ministry of Health and Welfare 2016. ^4^ The diagnostic values were defined by Okorodudu et al. [22]. ^5^ The diagnostic values were defined by the Dietitians Association of Australia 2009. ^6^ The diagnostic values were defined by the American Diabetes Association 2014. ^7^ The diagnostic values were defined in the executive summary of the Third Report of the National Cholesterol Education Program by the Expert Panel on Detection, Evaluation, and Treatment of High Blood Cholesterol in Adults 2001. ^8^ The diagnostic values were defined by the National Kidney Foundation’s Kidney Disease Outcomes Quality Initiative Work Group 2020. ^9^ The diagnostic values were defined by Ridker et al. [23]. ^10^ The diagnostic values were defined by the Taiwan Guideline for the Management of Gout and Hyperuricemia 2016. ^11^ The diagnostic values were defined by Kovesdy et al. [24].

**Table 2:** Dietary nutrient intake and the proportions of individuals within recommended target ranges (*n* = 106).

Nutrient	All Patients Mean ± SD		Patients within Normal Ranges *n* (%)		Recommended Dietary Intake Values
Calorie density, kcal/kg BW ^1^	30.41	7.2	29	(27.4)	≥35
Protein density, g/kg BW ^2^	1.12	0.3	19	(18.0)	M ≤ 0.84; F ≤ 0.75
Dietary carbohydrates, % ^3^	211.5	49.7	22	(20.8)	>50
Total dietary fat, % of energy ^2^	39.8	5.9	23	(21.7)	<35
SFA, % of energy ^2^	8.7	3.2	44	(41.5)	≤8
MUFA, % of energy ^2^	12.0	4.4	101	(95.3)	≤20
PUFA, % of energy ^3^	12.0	5.3	41	(38.7)	≤10
Cholesterol, mg ^3^	249.5	125.3	43	(40.6)	<200
Fiber, g ^2^	12.7	5.3	3	(2.8)	M ≥ 30; F ≥ 25
Vitamins					
Vitamin A, μg ^4^	778.4	517.3	53	(50.0)	≥700
Vitamin E, mg ^1^	28.8	15.2	21	(19.8)	>12
Vitamin C, mg ^4^	101.9	71.9	63	(59.4)	≥75
Vitamin B_1_, mg ^4^	1.0	0.4	33	(31.1)	M ≥1.2; F ≥ 1.1
Vitamin B_2_, mg ^4^	0.8	0.3	14	(13.2)	M ≥1.3; F ≥ 1.1
Vitamin B_3_ (Niacin), mg ^4^	11.6	4.3	26	(24.5)	M ≥ 16; F ≥ 14
Vitamin B_6_, mg ^4^	1.2	0.5	29	(27.4)	≤50 year ≥ 1.3; >50 year M ≥ 1.7; F ≥ 1.5
Vitamin B_12_, μg ^4^	2.9	2.2	50	(47.2)	≥2.4
Folic acid, μg ^4^	195.9	98.9	5	(4.7)	≥400
Minerals					
Sodium, mg ^2^	1020.9	655.5	10	(9.4)	≥2000
Potassium, mg ^4^	1822.6	619.0	40	(37.7)	≥1950
Calcium, mg ^2^	349.8	160.2	1	(0.9)	≥1000
Magnesium, mg ^1^	280.2	539.0	6	(6.6)	M ≥ 350; F ≥ 300
Phosphorus, mg ^4^	731.6	226.2	41	(38.7)	≥800
Iron, mg ^4^	8.4	2.8	31	(29.2)	M ≥ 8; F ≥ 15
Zinc, mg ^4^	8.1	2.8	3	(2.8)	M > 15; F > 12

M, male; F, female; SFA, saturated fatty acid; MUFA, monounsaturated fatty acid; PUFA, polyunsaturated fatty acid. ^1^ Recommended target values for dietary reference intakes by the Taiwan Ministry of Health and Welfare 2016. ^2^ Target values recommended by the Dietitians Association of Australia 2009. ^3^ Target values recommended under the National Kidney Foundation’s Kidney Disease Outcomes Quality Initiative 2020. ^4^ Target values outlined in the European Renal Best Practice Guidelines 2007. Data are expressed as means ± standard deviations or percentages as appropriate.

**Table 3:** Odds ratios of having traditional cardiovascular disease risk factors among renal transplant recipients with adequate nutrient intake.

	High WC	High BMI	High TBF	High BP	High FPG	HOMA	High TC	High LDL-C	Low HDL-C	High TG
	OR (95% CI)	(95% CI)	(95% CI)	(95% CI)	(95% CI)	(95% CI)	(95% CI)	(95% CI)	(95% CI)	(95% CI)
Nutrients										
Energy/kg			1.05 (0.12–9.30)	1.29 (0.15–11.19)	0.73 (0.27–2.00)	0.05 (0.01–0.81) ^†^	0.42 (0.04–4.30)	0.39 (0.04–3.93)	0.09 (0.01–1.28)	0.07 (0.00–1.25)
Protein/kg	1.08 (0.95–1.24)		1.07 (0.92–1.24)		0.85 (0.24–3.08)	1.02 (0.89–1.17)	1.04 (0.90–1.20)	1.09 (0.94–1.25)	0.87 (0.74–1.03)	0.98 (0.84–1.15)
Carbohydrate	1.12 (0.96–1.30)	1.04 (0.91–1.20)	1.08 (0.93–1.25)	1.08 (0.93–1.25)	0.53 (0.17–1.67)	1.03 (0.89–1.19)	0.99 (0.86–1.14)	0.90 (0.77–1.04)	1.10 (0.93–1.30)	1.26 (1.01–1.57) ^†^
Fat	0.94 (0.75–1.17)	0.89 (0.71–1.10)	0.98 (0.79–1.21)	0.98 (0.78–1.21)	0.65 (0.21–2.02)	1.07 (0.85–1.35)	1.00 (0.81–1.24)	0.91 (0.73–1.14)	1.09 (0.86–1.38)	1.16 (0.89–1.50)
SFA	0.95 (0.80–1.13)	0.94 (0.80–1.12)	1.04 (0.88–1.23)	0.98 (0.82–1.16)	0.42 (0.16–1.10)	1.15 (0.94–1.41)	0.93 (0.78–1.12)	0.87 (0.72–1.05)	1.13 (0.93–1.36)	1.11 (0.92–1.34)
PUFA	1.00 (1.00–1.01)	1.00 (1.00–1.01)	1.00 (1.00–1.01)	1.00 (0.99–1.01)	0.93 (0.36–2.41)	1.00 (0.99–1.00)	1.00 (1.00–1.01)	1.00 (1.00–1.01)	1.00 (0.99–1.01)	1.01 (1.00–1.02)
Cholesterol	1.00 (0.84–1.20)	1.11 (0.93–1.32)	1.03 (0.87–1.23)	1.01 (0.85–1.20)	1.33 (0.52–3.45)	1.18 (0.96–1.46)	1.06 (0.89–1.27)	0.98 (0.82–1.17)	1.02 (0.83–1.25)	0.91 (0.73–1.12)
Vitamins										
Vitamin A	1.09 (0.88–1.35)	1.15 (0.93–1.41)	1.30 (1.00–1.69)	1.07 (0.87–1.30)	1.06 (0.42–2.66)	1.22 (0.96–1.56)	0.69 (0.52–0.91) ^‡^	0.89 (0.72–1.1)	1.17 (0.91–1.51)	0.98 (0.79–1.22)
Vitamin C	0.46 (0.12–1.85)	0.48 (0.12–1.9)	0.67 (0.16–2.82)	1.83 (0.44–7.56)	0.63 (0.24–1.63)	2.68 (0.64–11.3)	1.04 (0.25–4.27)	0.88 (0.22–3.47)	0.92 (0.20–4.28)	2.80 (0.47–16.66)
Vitamin E	1.01 (0.99–1.02)	1.01 (0.99–1.02)	1.00 (0.99–1.01)	1.01 (1.00–1.03)	4.17 (1.04–16.73)	1.00 (0.99–1.02)	0.99 (0.98–1.01)	1.00 (0.99–1.02)	0.98 (0.96–1.00) ^†^	0.99 (0.97–1.00)
Vitamin B_1_	0.11 (0.01–1.03)	0.37 (0.06–2.44)	1.05 (0.16–6.87)	0.64 (0.08–4.93)	0.97 (0.35–2.72)	0.67 (0.10–4.44)	0.77 (0.12–4.83)	2.25 (0.21–23.95)	2.25 (0.21–23.95)	1.20 (0.14–10.25)
Vitamin B_2_	0.96 (0.81–1.12)	1.05 (0.89–1.22)	1.03 (0.88–1.21)		0.92 (0.24–3.57)	0.99 (0.84–1.17)	1.09 (0.92–1.29)	0.96 (0.80–1.16)	0.96 (0.80–1.16)	1.10 (0.90–1.33)
Niacin	2.70 (0.60–12.17)	1.79 (0.42–7.69)	1.97 (0.44–8.74)	1.94 (0.43–8.72)	0.69 (0.23–2.05)	1.92 (0.41–9.00)	0.64 (0.13–3.20)	0.24 (0.04–1.48)	0.24 (0.04–1.48)	7.31 (0.80–66.53)
Vitamin B_6_	0.90 (0.64–1.25)	1.06 (0.76–1.47)	1.15 (0.79–1.67)	0.88 (0.61–1.28)	1.00 (0.99–1.01)	0.75 (0.47–1.18)	0.90 (0.63–1.28)	0.75 (0.45–1.25)	1.13 (0.71–1.79)	1.73 (0.82–3.66)
Vitamin B_12_	1.00 (0.99–1.00)	1.00 (1.00–1.01)	1.00 (1.00–1.01)	1.00 (0.99–1.01)	0.49 (00.19–1.27)	1.00 (0.99–1.01)	1.00 (0.99–1.01)	1.00 (1.00–1.01)	1.00 (0.99–1.01)	1.01 (0.99–1.03)
Minerals										
Sodium	1.00 (1.00–1.00)				0.70 (0.15–3.25)			1.00 (1.00–1.00)		
Potassium	1.00 (1.00–1.01)	1.00 (1.00–1.01)	1.01 (1.00–1.01)	1.00 (0.99–1.00)	0.58 (0.22–1.50)	1.01 (1.00–1.01)	1.00 (1.00–1.01)	1.00 (1.00–1.01)	1.01 (1.00–1.01)	1.00 (0.99–1.00)
Phosphate	1.03 (0.82–1.29)	1.08 (0.87–1.35)	1.07 (0.86–1.34)	1.08 (0.86–1.35)	1.74 (0.67–4.54)	1.11 (0.87–1.42)	1.14 (0.91–1.44)	1.02 (0.81–1.28)	1.21 (0.91–1.60)	1.07 (0.83–1.37)
Iron	1.02 (0.83–1.24)	1.12 (0.92–1.37)	1.09 (0.89–1.34)	1.11 (0.90–1.37)	1.04 (0.33–3.33)	0.96 (0.79–1.17)	1.07 (0.88–1.30)	1.04 (0.85–1.26)	1.06 (0.84–1.32)	1.02 (0.83–1.27)

OR, odds ratio; CI, confidence interval; WC, waist circumference; BMI, body mass index; TBF, total body fat; BP; blood pressure; FPG, fasting plasma glucose; HOMA, homeostasis model assessment; TC, total cholesterol; LDL-C, low-density lipoprotein cholesterol; HDL-C, high-density lipoprotein cholesterol; TG, triglyceride; SFA, saturated fatty acid; PUFA, polyunsaturated fatty acids. The analysis was adjusted for age, gender, post-renal transplant vintage, glomerular filtration rate and Charlson comorbidity index. Significant level at ^†^ *p* < 0.05, ^‡^ *p* < 0.01. Nutrients with insufficient number of subjects for analysis: monounsaturated fatty acids, fiber, calcium, magnesium, zinc, and folic acid. Blank: insufficient subjects for analysis.

**Table 4:** Odds ratios of having non-traditional cardiovascular disease risk factors among renal transplant recipients with adequate nutrient intake.

	Anemia	Low eGFR OR (95% CI)	Low Ca	Low P	High K	High UA	High hs-CRP	High iPTH
	OR (95% CI)		OR (95% CI)	OR (95% CI)	OR (95% CI)	OR (95% CI)	OR (95% CI)	OR (95% CI)
Nutrients								
Energy/kg	2.99 (0.26–34.78)	1.42 (0.17–11.61)		2.49 (0.27–23.03)		0.42 (0.04–4.92)		0.63 (0.04–10.40)
Protein/kg	0.89 (0.75–1.05)	1.02 (0.89–1.18)		0.96 (0.84–1.10)		1.32 (1.03–1.70) ^†^		
CHO		1.03 (0.90–1.18)		0.91 (0.78–1.05)		0.95 (0.82–1.1)	0.97 (0.74–1.28)	
Fat	1.03 (0.82–1.28)	0.96 (0.77–1.19)		1.04 (0.83–1.31)		0.92 (0.73–1.16)	0.86 (0.57–1.30)	1.01 (0.78–1.32)
SFA	1.10 (0.92–1.32)	0.98 (0.83–1.16)		1.06 (0.89–1.27)	1.00 (0.79–1.28)	0.97 (0.81–1.16)	1.04 (0.77–1.39)	1.10 (0.89–1.35)
PUFA	1.00 (1.00–1.01)	1.00 (0.99–1.01)		0.99 (0.99–1.00) ^†^	1.00 (0.99–1.00)	1.00 (0.99–1.00)	1.01 (1.00–1.02)	1.00 (1.00–1.01)
Cholesterol	1.24 (1.00–1.54)	1.00 (0.84–1.18)	0.75 (0.50–1.11)	1.00 (0.83–1.20)		0.88 (0.73–1.07)	1.21 (0.86–1.71)	1.11 (0.87–1.42)
Vitamins								
Vitamin A	2.10 (0.45–9.81)	1.09 (0.89–1.33)	1.05 (0.70–1.57)	1.14 (0.93–1.39)		1.06 (0.86–1.32)	1.45 (0.97–2.17)	1.34 (0.92–1.96)
Vitamin C	2.10 (0.45–9.81)	0.30 (0.07–1.25)	0.96 (0.09–10.73)	0.44 (0.11–1.86)		0.95 (0.18–5.06)	0.14 (0.00–5.26)	0.70 (0.11–4.32)
Vitamin E	0.99 (0.98–1.01)	1.00 (0.99–1.01)		1.01 (0.99–1.02)	0.99 (0.97–1.01)	1.01 (0.99–1.02)	1.02 (1.00–1.05)	
Vitamin B_1_	1.85 (0.24–14.29)	0.61 (0.25–1.50)		0.56 (0.09–3.64)	0.61 (0.02–20.01)	2.06 (0.19–22.99)	1.71 (0.03–117.92)	1.64 (0.11–25.64)
Vitamin B_2_	1.23 (1.01–1.50) ^†^	0.85 (0.25–2.95)		0.84 (0.70–1.01)				
Niacin	0.93 (0.19–4.58)	0.56 (0.21–1.49)		1.54 (0.33–7.22)		0.46 (0.08–2.66)	1.14 (0.09–14.16)	
Vitamin B_6_	0.86 (0.61–1.21)	1.40 (0.83–2.37)		1.17 (0.83–1.66)	0.89 (0.60–1.33)	1.05 (0.70–1.60)	0.85 (0.38–1.89)	
Vitamin B_12_	1.00 (0.99–1.00)	1.00 (1.00–1.01)	1.01 (0.99–1.03)	1.00 (1.00–1.01)		1.00 (0.99–1.00)	1.00 (0.99–1.02)	1.01 (1.00–1.03)
Minerals								
Sodium	1.00 (1.00–1.00)							
Potassium	1.01 (1.00–1.01)	1.00 (1.00–1.01)	1.00 (0.99–1.01)	1.00 (0.99–1.00)		1.00 (0.99–1.00)	1.00 (0.99–1.01)	1.00 (1.00–1.01)
Phosphate	1.33 (0.98–1.80)	0.92 (0.74–1.14)		0.83 (0.62–1.09)	0.82 (0.58–1.14)	0.66 (0.46–0.94) ^†^	0.90 (0.55–1.47)	
Iron	1.34 (1.01–1.80) ^†^	1.02 (0.84–1.24)		0.78 (0.59–1.03)		0.81 (0.64–1.03)		

OR, odds ratio; CI, confidence interval; Alb, albumin; Cr, creatinine; eGFR, estimated Glomerular filtration rate; Ca, calcium; P, phosphorus; K, potassium; UA, uric acid; hs-CRP, high sensitivity C-reactive protein; iPTH, intact parathyroid hormone; CHO, carbohydrate; SFA, saturated fatty acid; PUFA, polyunsaturated fatty acids. The analysis was adjusted for age, gender, post-renal transplant vintage, glomerular filtration rate, and Charlson comorbidity index. Significant level at † *p* < 0.05. Nutrients with insufficient number of subjects for analysis: monounsaturated fatty acids, fiber, calcium, magnesium, zinc, and folic acid. Blank: insufficient subjects for analysis.

## Data Availability

The original contributions presented in the study are included in the article/supplementary material; further inquiries can be directed to the corresponding author.

## Supplementary Materials

The following are available online at https://www.mdpi.com/article/10.3390/ijerph18168448/s1, Table S1: Proportion of nutrient intake with traditional cardiovascular risk factors among renal transplant recipients (*n* = 106), Table S2: Proportion of nutrient intake with nontraditional cardiovascular risk factors among renal transplant recipients (*n* = 106).
