# Supplementary material for: Dietary Nutrients and Cardiovascular Risk Factors among Renal Transplant Recipients
Source: Int J Environ Res Public Health. 2021 Aug 10;18(16):8448. doi: 10.3390/ijerph18168448 (PMC8391485; doi:10.3390/ijerph18168448)
Supplement: Supplementary file 1 [file ijerph-18-08448-s001.zip › ijerph-1319188-supplementary.pdf]

Table S1. Proportion of nutrients intake with having traditional cardiovascular risk factors among renal transplant recipients (n = 106).

	WC				BMI				TBF				High BP				High FPG				HOMA				High TC				High LDL-C				Low HDL-C				High TG			
	Normal		High		Normal		High		Normal		High		Normal		High		Normal		High		Normal		High		Normal		High		Normal		High		Low		Normal		Normal		High	
n	62		44		51		55		51		54		38		68		94		12		50		53		52		54		37		69		65		41		74		32	
	n	%	n	%	n	%	n	%	n	%	n	%	n	%	n	%	n	%	n	%	n	%	n	%	n	%	n	%	n	%	n	%	n	%	n	%	n	%	n	%
**Nutrients**																																								
Energy, kcal/kg																																								
High (> 35)	26	41.9			28	54.9			19	37.3	9	16.7	12	31.6	17	25.0	27	28.7			17	34.0	12	22.6	15	28.9	14	25.9	11	29.7	18	26.1	18	27.7	11	26.8	21	28.4	8	25.0
Adequate (≤ 35)	36	58.1			23	45.1			32	62.7	45	83.3	26	68.4	51	75.0	67	71.3			33	66.0	41	77.4	37	71.2	40	74.1	26	70.2	51	73.9	47	72.3	30	73.2	53	71.6	24	75.0
Protein, g/kg BW																																								
High (M > 0.84; F > 0.75)	57	91.9	28	63.6			38	69.1	43	84.3	41	75.9			51	75.0	76	80.9			38	76.0	44	83.0	45	86.5	40	74.1	32	86.5	53	76.8	52	80.0	33	80.5	61	82.4	24	75.0
Adequate (M: ≤ 0.84; F ≤ 0.75)	5	8.1	16	36.4			17	30.9	8	15.7	13	24.1			17	25.0	18	19.1			12	24.0	9	17.0	7	13.5	14	25.9	5	13.5	16	23.2	13	20.0	8	19.5	13	17.6	8	25.0
Carbohydrates, % of energy																																								
Adequate (> 50)	12	19.4	10	22.7	8	15.7	14	25.5	9	17.6	13	24.1	8	21.1	14	20.6	17	18.1	5	41.7	8	16.0	14	26.4	12	23.1	10	18.5	11	29.7	11	15.9	7	10.8	15	36.6	13	17.6	9	28.1
Inadequate (≤ 50)	50	80.6	34	77.3	43	84.3	41	74.5	42	82.4	41	75.9	30	78.9	54	79.4	77	81.9	7	58.3	42	84.0	39	73.6	40	76.9	44	81.5	26	70.3	58	84.1	58	89.2	26	63.4	61	82.4	23	71.9
Total dietary fat, % of energy																																								
High (> 35)	51	82.3	32	72.7	42	82.4	41	74.5	45	88.2	37	68.5	31	81.6	52	76.5	75	79.8			40	80.0	40	75.5	41	78.9	42	77.8	27	73.0	56	81.2	57	87.7	26	63.4	61	82.4	22	68.8
Adequate (≤ 35)	11	17.7	12	27.3	9	17.6	14	25.5	6	11.8	17	31.5	7	18.4	16	23.5	19	20.2			10	20.0	13	24.5	11	21.2	12	22.2	10	27.0	13	18.8	8	12.3	15	36.6	13	17.6	10	31.3
SFAs, % of energy																																								
Adequate (≤ 8)	28	45.2	16	36.4	21	41.2	23	41.8	22	43.1	22	40.7	17	44.7	27	39.7	39	41.5	5	41.7	21	42.0	20	37.7	22	42.3	22	40.7	18	48.7	26	37.7	29	44.6	15	36.6	32	43.2	12	37.5
High (> 8)	34	54.8	28	63.6	30	58.8	32	58.2	29	56.9	32	59.3	21	55.3	41	60.3	55	58.5	7	58.3	29	58.0	33	62.3	30	57.7	32	59.3	19	51.4	43	62.3	36	55.4	26	63.4	42	56.8	20	62.5
PUFAs, % of energy																																								
Adequate (≤ 10)	27	43.5	14	31.8	17	33.3	24	43.6	21	41.2	20	37.0	17	44.7	24	35.3	34	36.2	7	58.3	18	36.0	21	39.6	15	28.9	26	48.2	12	32.4	29	42.0	25	38.5	16	39.0	28	37.8	13	40.6
High (> 10)	35	56.5	30	68.2	34	66.7	31	56.4	30	58.8	34	63.0	21	55.3	44	64.7	60	63.8	5	41.7	32	64.0	32	60.4	37	71.2	28	51.9	25	67.6	40	58.0	40	61.5	25	61.0	46	62.2	19	59.4
Cholesterol, mg																																								
Adequate (< 200)	25	40.3	18	40.9	21	41.2	22	40.0	19	37.3	24	44.4	16	42.1	27	39.7	37	39.4	6	50.0	19	38.0	24	45.3	25	48.1	18	33.3	17	46.0	26	37.7	27	41.5	16	39.0	26	35.1	17	53.1
High (≥ 200)	37	59.7	26	59.1	30	58.8	33	60.0	32	62.7	30	55.6	22	57.9	41	60.3	57	60.6	6	50.0	31	62.0	29	54.7	27	51.9	36	66.7	20	54.1	43	62.3	38	58.5	25	61.0	48	64.9	15	46.9
**Minerals**																																								
Sodium, mg																																								
Adequate (≥ 2000)	5	8.1	5	11.4			7	12.7			6	11.1			6	8.8	8	8.5							7	13.5			5	13.5	5	7.3	6	9.2			9	12.2		
Inadequate (< 2000)	57	91.9	39	88.6			48	87.3			48	88.9			62	91.2	86	91.5							45	86.5			32	86.5	64	92.8	59	90.8			65	87.8		
Potassium, mg																																								
Adequate (≥ 1950)	23	37.1	17	38.6	23	45.1	17	30.9	19	37.3	20	37.0	13	34.2	27	39.7	37	39.4			23	46.0	17	32.1	22	42.3	18	33.3	15	40.5	25	36.2	26	40.0	14	34.2	30	40.5	10	31.3
Inadequate (< 1950)	39	62.9	27	61.4	28	54.9	38	69.1	32	62.7	34	63.0	25	65.8	41	60.3	57	60.6			27	54.0	36	67.9	30	57.7	36	66.7	22	59.5	44	63.8	39	60.0	27	65.9	44	59.5	22	68.8
Phosphate, mg																																								
Adequate (≥ 800)	27	43.5	14	31.8	25	49.0	6	10.9	22	43.1	18	33.3	16	42.1	25	36.8	37	39.4			19	38.0	19	35.9	20	38.5	21	38.9	15	40.5	26	37.7	24	36.9	17	41.5	29	39.2	12	37.5
Inadequate (< 800)	35	56.5	30	68.2	26	51.0	49	89.1	29	56.9	36	66.7	22	57.9	43	63.2	57	60.6			31	62.0	34	64.2	32	61.5	33	61.1	22	59.5	43	62.3	41	63.1	24	58.5	45	60.8	20	62.5
Iron, mg									0																															
Adequate (M ≥ 8; F ≥ 15)	22	35.5	9	20.5	14	27.5	17	30.9	20	39.2	9	16.7	13	34.2	18	26.5	28	29.8			14	28.0	15	28.3	16	30.8	15	27.8	10	27.0	21	30.4	23	35.4	8	19.5	20	27.0	11	34.4
Inadequate (M < 8; F < 15)	40	64.5	35	79.5	37	72.5	38	69.1	31	60.8	45	83.3	25	65.8	50	73.5	66	70.2			36	72.0	38	71.7	36	69.2	39	72.2	27	73.0	48	69.6	42	64.6	33	80.5	54	73.0	21	65.6
**Vitamins**																																								
Vitamin A, μg																																								
Adequate (≥ 700)	30	48.4	23	52.3	26	51.0	27	49.1	23	45.1	30	55.6	22	57.9	31	45.6	47	50.0	6	50.0	25	50.0	25	47.2	24	46.2	29	53.7	16	43.2	37	53.6	33	50.8	20	48.8	39	52.7	14	43.8
Inadequate (< 700)	32	51.6	21	47.7	25	49.0	28	50.9	28	54.9	24	44.4	16	42.1	37	54.4	47	50.0	6	50.0	25	50.0	28	52.8	28	53.9	25	46.3	21	56.8	32	46.4	32	49.2	21	51.2	35	47.3	18	56.3
Vitamin E, mg																																								
Adequate (> 12)	14	22.6	7	15.9	13	25.5	8	14.5	13	25.5	8	14.8	8	21.1	13	19.1	19	20.2			10	20.0	11	20.8	10	19.2	11	20.4	9	24.3	12	17.4	13	20.0	8	19.5	16	21.6	5	15.6

Inadequate (≤ 12)	48	77.4	37	84.1	38	74.5	47	85.5	38	74.5	46	85.2	30	78.9	55	80.9	75	79.8		
Vitamin C, mg																				
Adequate (≥ 75)	39	62.9	24	54.5	34	66.7	29	52.7	31	60.8	31	57.4	25	65.8	38	55.9	54	57.4		
Inadequate (< 75)	23	37.1	20	45.5	17	33.3	26	47.3	20	39.2	23	42.6	13	34.2	30	44.1	40	42.6		
Vitamin B_1, mg																				
Inadequate (M < 1.2; F < 1.1)	21	33.9	12	27.3	21	41.2	12	21.8	18	35.3	14	25.9	12	31.6	21	30.9	29	30.9		
Adequate (M ≥1.2; F ≥ 1.1)	41	66.1	32	72.7	30	58.8	43	78.2	33	64.7	40	74.1	26	68.4	47	69.1	65	69.1		
Vitamin B_2, mg																				
Adequate (M ≥1.3; F ≥ 1.1)	7	11.3	7	15.9	8	15.7	6	10.9	9	17.6	5	9.3			10	14.7	14	14.9		
Inadequate (M < 1.3; F < 1.1)	55	88.7	37	84.1	43	84.3	49	89.1	42	82.4	49	90.7			58	85.3	80	85.1		
Niacin, mg																				
Adequate (M ≥ 16; F ≥ 14)	18	29.0	8	18.2	16	31.4	10	18.2	14	27.5	11	20.4	10	26.3	16	23.5	22	23.4		
Inadequate (M < 16; F < 14)	44	71.0	36	81.8	35	68.6	45	81.8	37	72.5	43	79.6	28	73.7	52	76.5	72	76.6		
Vitamin B_6, mg																				
Adequate (≤ 50 y: ≥1.3; > 50 y: M ≥1.7; F ≥1.5)	20	32.3	9	20.5	19	37.3	10	18.2	18	35.3	10	18.5	11	28.9	18	26.5	25	26.6		
Inadequate (≤ 50 y: <1.3; > 50 y: M <1.7; F <1.5)	42	67.7	35	79.5	32	62.7	45	81.8	33	64.7	44	81.5	27	71.1	50	73.5	69	73.4		
Vitamin B_{12}, mg/d																				
Adequate (≥ 2.4)	30	48.4	20	45.5	23	45.1	27	49.1	27	52.9	23	42.6	23	60.5	27	39.7	44	46.8	6	50.0
Inadequate (< 2.4)	32	51.6	24	54.5	28	54.9	28	50.9	24	47.1	31	57.4	15	39.5	41	60.3	50	53.2	6	50.0

Inadequate (≤ 12)	40	80.0	42	79.3	42	80.8	43	79.6	28	75.7	57	82.6	52	80.0	33	80.5	58	78.4	27	84.4
Vitamin C, mg																				
Adequate (≥ 75)	29	58.0	32	60.4	33	63.5	30	55.6	26	70.3	37	53.6	33	50.8	30	73.2	44	59.5	19	59.4
Inadequate (< 75)	21	42.0	21	39.6	19	36.5	24	44.4	11	29.7	32	46.4	32	49.2	11	26.8	30	40.5	13	40.6
Vitamin B_1, mg																				
Inadequate (M < 1.2; F < 1.1)	12	24.0	21	39.6	18	34.6	15	27.8	12	32.4	21	30.4	20	30.8	13	31.7	23	31.1	10	31.3
Adequate (M ≥1.2; F ≥ 1.1)	38	76.0	32	60.4	34	65.2	39	72.2	25	67.6	48	69.6	45	69.2	28	68.3	51	68.9	22	68.8
Vitamin B_2, mg																				
Adequate (M ≥1.3; F ≥ 1.1)	6	12.0	8	15.1	8	15.4	6	11.1	5	13.5	9	13.0	9	13.9	5	12.2	9	12.2	5	15.6
Inadequate (M < 1.3; F < 1.1)	44	88.0	45	84.9	44	84.6	48	88.9	32	86.5	60	87.0	56	86.2	36	87.8	65	87.8	27	84.4
Niacin, mg																				
Adequate (M ≥ 16; F ≥ 14)	9	18.0	16	30.2	15	28.9	11	20.4	9	24.3	17	24.6	14	21.5	12	29.3	18	24.3	8	25.0
Inadequate (M < 16; F < 14)	41	82.0	37	69.8	37	71.2	43	79.6	28	75.7	52	75.4	51	78.5	29	70.7	56	75.7	24	75.0
Vitamin B_6, mg																				
Adequate (≤ 50 y: ≥1.3; > 50 y: M ≥1.7; F ≥1.5)	14	28.0	15	28.3	14	26.9	15	27.8	8	21.6	21	30.4	15	23.1	14	34.2	22	29.7	7	21.9
Inadequate (≤ 50 y: <1.3; > 50 y: M <1.7; F <1.5)	36	72.0	38	71.7	38	73.1	39	72.2	29	78.4	48	69.6	50	76.9	27	65.9	52	70.3	25	78.1
Vitamin B_{12}, mg/d																				
Adequate (≥ 2.4)	22	44.0	28	52.8	27	51.9	23	42.6	18	48.7	32	46.4	27	41.5	23	56.1	36	48.7	14	43.8
Inadequate (< 2.4)	28	56.0	25	47.2	25	48.1	31	57.4	19	51.4	37	53.6	38	58.5	18	43.9	38	51.4	18	56.3

OR, odds ratio; CI, confidence interval; WC, waist circumference; BMI, body mass index; TBF, total body fat; BP, blood pressure; FPG, fasting plasma glucose; HOMA, homeostasis model assessment; TC, total cholesterol; LDL-C, low-density lipoprotein cholesterol; HDL-C, high-density lipoprotein cholesterol; TG, triglyceride; SFAs, saturated fatty acids; PUFAs, polyunsaturated fatty acids; M, male; F, female; y, years.

The analysis was adjusted for age, gender, post renal transplant vintage, glomerular filtration rate and Charlson comorbidity index. Significant level at $^{\dagger}P < 0.05$, $^{\ddagger}P < 0.01$.

Nutrients of which the intake was inadequate: monounsaturated fatty acids, dietary fiber, calcium, magnesium, zinc, and folic acid

Blank: inadequate subjects to analysis

Table S2. Proportion of nutrients intake with having non-traditional cardiovascular risk factors among renal transplant recipients (n = 106).

	Anemia				Low Alb				Low eGFR				Low Ca				Low P				High K				High UA				High hs-CRP				High iPTH			
	Low		Normal		Low		Normal		Low		Normal		Low		Normal		Low		Normal		Normal		High		Normal		High		Normal		High		Normal		High	
n	51		55		53		53		33		73		11		95		57		42		93		12		70		36		19		87		79		18	
	n	%	n	%	n	%	n	%	n	%	n	%	n	%	n	%	n	%	n	%	n	%	n	%	n	%	n	%	n	%	n	%	n	%	n	%
**Nutrients**																																				
Energy, kcal/kg																																				
High (> 35)	15	29.4	14.0	25.5	14.0	26.4	15.0	28.3	8.0	24.2	21.0	28.8		36.4	25.0	26.3	19.0	33.3	9.0	21.4	25.0	26.9		33.3	17.0	24.3	12.0	33.3		21.1	25.0	28.7	20.0	25.3	7.0	38.9
Adequate (≦ 35)	36	70.6	41.0	74.5	39.0	73.6	38.0	71.7	25.0	75.8	52.0	71.2	7.0	63.6	70.0	73.7	38.0	66.7	33.0	78.6	68.0	73.1	8.0	66.7	53.0	75.7	24.0	66.7	15.0	78.9	62.0	71.3	59.0	74.7	11.0	61.1
Protein, g/kg BW																																				
High (M > 0.84; F > 0.75)	40	78.4	45.0	81.8	47.0	88.7	39.0	73.6	27.0	81.8	59.0	80.8	10.0	90.9	76.0	80.0	50.0	87.7	30.0	71.4	76.0	81.7	9.0	75.0	56.0	80.0	30.0	83.3	15.0	78.9	71.0	81.6	62.0	78.5	16.0	88.9
Adequate (M: ≤ 0.84; F ≤ 0.75)	11	21.6	10.0	18.2	6.0	11.3	14.0	26.4	6.0	18.2	14.0	19.2		9.1	19.0	20.0	7.0	12.3	12.0	28.6	17.0	18.3		25.0	14.0	20.0	6.0	16.7		21.1	16.0	18.4	17.0	21.5		11.1
Carbohydrates, % of energy																																				
Adequate (> 50)	4	7.8	10.0	18.2	11.0	20.8	11.0	20.8	8.0	24.2	14.0	19.2		18.2	20.0	21.1	9.0	15.8	11.0	26.2	20.0	21.5		16.7	16.0	22.9	6.0	16.7	5.0	26.3	17.0	19.5	16.0	20.3		16.7
Inadequate (≤ 50)	47	92.2	45.0	81.8	42.0	79.2	42.0	79.2	25.0	75.8	59.0	80.8	9.0	81.8	75.0	78.9	48.0	84.2	31.0	73.8	73.0	78.5	10.0	83.3	54.0	77.1	30.0	83.3	14.0	73.7	70.0	80.5	63.0	79.7	15.0	83.3
Total dietary fat, % of energy																																				
High (> 35)	37	72.5	46.0	83.6	40.0	75.5	43.0	81.1	26.0	78.8	57.0	78.1	9.0	81.8	74.0	77.9	48.0	84.2	31.0	73.8	73.0	78.5	9.0	75.0	54.0	77.1	29.0	80.6	14.0	73.7	69.0	79.3	64.0	81.0	13.0	72.2
Adequate (≤ 35)	14	27.5	9.0	16.4	13.0	24.5	10.0	18.9	7.0	21.2	16.0	21.9		18.2	21.0	22.1	9.0	15.8	11.0	26.2	20.0	21.5		25.0	16.0	22.9	7.0	19.4	5.0	26.3	18.0	20.7	15.0	19.0	5.0	27.8
SFAs, % of energy																																				
Adequate (≤ 8)	25	49.0	20.0	36.4	19.0	35.8	25.0	47.2	13.0	39.4	31.0	42.5		27.3	41.0	43.2	22.0	38.6	17.0	40.5	37.0	39.8	6.0	50.0	29.0	41.4	15.0	41.7	10.0	52.6	34.0	39.1	29.0	36.7	6.0	33.3
High (> 8)	26	51.0	35.0	63.6	34.0	64.2	28.0	52.8	20.0	60.6	42.0	57.5	8.0	72.7	54.0	56.8	35.0	61.4	25.0	59.5	56.0	60.2	6.0	50.0	41.0	58.6	21.0	58.3	9.0	47.4	53.0	60.9	50.0	63.3	12.0	66.7
PUFAs, % of energy																																				
Adequate (≤ 10)	21	41.2	35.0	63.6	22.0	41.5	19.0	35.8	12.0	36.4	29.0	39.7		18.2	39.0	41.1	22.0	38.6	15.0	35.7	35.0	37.6	5.0	41.7	23.0	32.9	18.0	50.0	8.0	42.1	33.0	37.9	28.0	35.4	8.0	44.4
High (> 10)	30	58.8	20.0	36.4	31.0	58.5	34.0	64.2	21.0	63.6	44.0	60.3	9.0	81.8	56.0	58.9	35.0	61.4	27.0	64.3	58.0	62.4	7.0	58.3	47.0	67.1	18.0	50.0	11.0	57.9	54.0	62.1	51.0	64.6	10.0	55.6
Cholesterol, mg																																				
Adequate (< 200)	24	47.1	19.0	34.5	21.0	39.6	22.0	41.5	14.0	42.4	29.0	39.7	6.0	54.5	37.0	38.9	20.0	35.1	21.0	50.0	39.0	41.9		25.0	29.0	41.4	14.0	38.9	8.0	42.1	35.0	40.2	28.0	35.4	11.0	61.1
High (≥ 200)	27	52.9	36.0	65.5	32.0	60.4	31.0	58.5	19.0	57.6	44.0	60.3	5.0	45.5	58.0	61.1	37.0	64.9	21.0	50.0	54.0	58.1	9.0	75.0	41.0	58.6	22.0	61.1	11.0	57.9	52.0	59.8	51.0	64.6	7.0	38.9
**Minerals**																																				
Sodium, mg	5																																			
Adequate (≥ 2000)	46	9.8	5.0	9.1	5.0	9.4	6.0	11.3	6.0	18.2		5.5		0.0	10.0	10.5	7.0	12.3		4.8	8.0	8.6		16.7		5.7	6.0	16.7		5.3	9.0	10.3	7.0	8.9		5.6
Inadequate (< 2000)		90.2	50.0	90.9	48.0	90.6	47.0	88.7	27.0	81.8	69.0	94.5	11.0	100.0	85.0	89.5	50.0	87.7	40.0	95.2	85.0	91.4	10.0	83.3	66.0	94.3	30.0	83.3	18.0	94.7	78.0	89.7	72.0	91.1	17.0	94.4
Potassium, mg																																				

Adequate (≥ 1950)	18	35.3	22.0	40.0	19.0	35.8	21.0	39.6	10.0	30.3	30.0	41.1	5.0	45.5	35.0	36.8	22.0	38.6	18.0	42.9	35.0	37.6		33.3	24.0	34.3	16.0	44.4	8.0	42.1	32.0	36.8	32.0	40.5	5.0	27.8
Inadequate (< 1950)	33	64.7	33.0	60.0	34.0	64.2	32.0	60.4	23.0	69.7	43.0	58.9	6.0	54.5	60.0	63.2	35.0	61.4	24.0	57.1	58.0	62.4	8.0	66.7	46.0	65.7	20.0	55.6	11.0	57.9	55.0	63.2	47.0	59.5	13.0	72.2
Phosphate, mg																																				
Adequate (≥ 800)	18	35.3	23.0	41.8	23.0	43.4	18.0	34.0	12.0	36.4	29.0	39.7		27.3	38.0	40.0	22.0	38.6	15.0	35.7	34.0	36.6	6.0	50.0	25.0	35.7	16.0	44.4	7.0	36.8	24.0	27.6	35.0	44.3		11.1
Inadequate (< 800)	33	64.7	32.0	58.2	30.0	56.6	35.0	66.0	21.0	63.6	44.0	60.3	8.0	72.7	57.0	60.0	35.0	61.4	27.0	64.3	59.0	63.4	6.0	50.0	45.0	64.3	20.0	55.6	12.0	63.2	63.0	72.4	44.0	55.7	16.0	88.9
Iron, mg																																				
Adequate (M ≥ 8; F ≥ 15)	11	21.6	20.0	36.4	16.0	30.2	15.0	28.3	13.0	39.4	18.0	24.7		18.2	29.0	30.5	16.0	28.1	11.0	26.2	20.0	21.5	10.0	83.3	15.0	21.4	16.0	44.4		21.1	27.0	31.0	23.0	29.1		16.7
Inadequate (M < 8; F < 15)	40	78.4	35.0	63.6	37.0	69.8	38.0	71.7	20.0	60.6	55.0	75.3	9.0	81.8	66.0	69.5	41.0	71.9	31.0	73.8	73.0	78.5		16.7	55.0	78.6	20.0	55.6	15.0	78.9	60.0	69.0	56.0	70.9	15.0	83.3
**Vitamins**																																				
Vitamin A, RE																																				
Adequate (≥ 700)	27	52.9	26.0	47.3	27.0	50.9	27.0	50.9	17.0	51.5	36.0	49.3	5.0	45.5	48.0	50.5	30.0	52.6	18.0	42.9	40.0	43.0	12.0	100.0	30.0	42.9	23.0	63.9	8.0	42.1	45.0	51.7	39.0	49.4	8.0	44.4
Inadequate (< 700)	24	47.1	29.0	52.7	26.0	49.1	26.0	49.1	16.0	48.5	37.0	50.7	6.0	54.5	47.0	49.5	27.0	47.4	24.0	57.1	53.0	57.0		0.0	40.0	57.1	13.0	36.1	11.0	57.9	42.0	48.3	40.0	50.6	10.0	55.6
Vitamin E, mg										0.0																										
Adequate (> 12)	12	23.5	9.0	16.4	8.0	15.1	13.0	24.5	6.0	18.2	15.0	20.5		18.2	19.0	20.0	16.0	28.1	5.0	11.9	16.0	17.2	5.0	41.7	16.0	22.9	5.0	13.9	6.0	31.6	15.0	17.2	17.0	21.5		16.7
Inadequate (≤ 12)	39	76.5	46.0	83.6	45.0	84.9	40.0	75.5	27.0	81.8	58.0	79.5	9.0	81.8	76.0	80.0	41.0	71.9	37.0	88.1	77.0	82.8	7.0	58.3	54.0	77.1	31.0	86.1	13.0	68.4	72.0	82.8	62.0	78.5	15.0	83.3
Vitamin C, mg																																				
Adequate (≥ 75)	29	56.9	34.0	61.8	33.0	62.3	30.0	56.6	16.0	48.5	47.0	64.4	5.0	45.5	58.0	61.1	32.0	56.1	27.0	64.3	53.0	57.0	9.0	75.0	43.0	61.4	20.0	55.6	12.0	63.2	51.0	58.6	49.0	62.0	9.0	50.0
Inadequate (< 75)	22	43.1	21.0	38.2	20.0	37.7	23.0	43.4	17.0	51.5	26.0	35.6	6.0	54.5	37.0	38.9	25.0	43.9	15.0	35.7	40.0	43.0		25.0	27.0	38.6	16.0	44.4	7.0	36.8	36.0	41.4	30.0	38.0	9.0	50.0
Vitamin B_1, mg																																				
Inadequate (M < 1.2; F < 1.1)	15	29.4	18.0	32.7	17.0	32.1	16.0	30.2	13.0	39.4	20.0	27.4		36.4	29.0	30.5	23.0	40.4	10.0	23.8	27.0	29.0	6.0	50.0	20.0	28.6	13.0	36.1	8.0	42.1	25.0	28.7	27.0	34.2	5.0	27.8
Adequate (M ≥1.2; F ≥ 1.1)	36	70.6	37.0	67.3	36.0	67.9	37.0	69.8	20.0	60.6	53.0	72.6	7.0	63.6	66.0	69.5	34.0	59.6	32.0	76.2	66.0	71.0	6.0	50.0	50.0	71.4	23.0	63.9	11.0	57.9	62.0	71.3	52.0	65.8	13.0	72.2
Vitamin B_2, mg																																				
Adequate (M ≥1.3; F ≥ 1.1)	6	11.8	8.0	14.5	8.0	15.1	6.0	11.3	5.0	15.2	9.0	12.3		36.4	10.0	10.5	9.0	15.8	8.0	19.0	11.0	11.8		16.7	11.0	15.7		8.3		15.8	11.0	12.6	11.0	13.9		11.1
Inadequate (M < 1.3; F < 1.1)	45	88.2	47.0	85.5	45.0	84.9	47.0	88.7	28.0	84.8	64.0	87.7	7.0	63.6	85.0	89.5	48.0	84.2	34.0	81.0	82.0	88.2	10.0	83.3	59.0	84.3	33.0	91.7	16.0	84.2	76.0	87.4	68.0	86.1	16.0	88.9
Niacin, mg																																				
Adequate (M ≥ 16; F ≥ 14)	8	15.7	18.0	32.7	14.0	26.4	12.0	22.6	11.0	33.3	18.0	24.7		18.2	24.0	25.3	19.0	33.3	6.0	14.3	22.0	23.7		33.3	17.0	24.3	9.0	25.0	5.0	26.3	21.0	24.1	23.0	29.1		11.1
Inadequate (M < 16; F < 14)	43	84.3	37.0	67.3	39.0	73.6	41.0	77.4	22.0	66.7	55.0	75.3	9.0	81.8	71.0	74.7	38.0	66.7	36.0	85.7	71.0	76.3	8.0	66.7	53.0	75.7	27.0	75.0	14.0	73.7	66.0	75.9	56.0	70.9	16.0	88.9
Vitamin B_6, mg																																				
Adequate (≤ 50 y: ≥1.3; > 50 y: M ≥1.7; F ≥1.5)	12	23.5	17.0	30.9	12.0	22.6	17.0	32.1	7.0	21.2	22.0	30.1		36.4	25.0	26.3	18.0	31.6	9.0	21.4	24.0	25.8	5.0	41.7	20.0	28.6	9.0	25.0	8.0	42.1	21.0	24.1	25.0	31.6		16.7

Inadequate (≤ 50 y: <1.3; > 50 y: M <1.7; F <1.5)	39	76.5	38.0	69.1	41.0	77.4	36.0	67.9	26.0	78.8	51.0	69.9	7.0	63.6	70.0	73.7	39.0	68.4	33.0	78.6	69.0	74.2	7.0	58.3	50.0	71.4	27.0	75.0	11.0	57.9	66.0	75.9	54.0	68.4	15.0	83.3
Vitamin B_{12}, mg/d																																				
Adequate (≥ 2.4)	24	47.1	26.0	47.3	26.0	49.1	24.0	45.3	17.0	51.5	33.0	45.2	5.0	45.5	45.0	409.1	29.0	50.9	19.0	45.2	41.0	44.1	8.0	66.7	33.0	47.1	17.0	47.2	8.0	42.1	42.0	48.3	40.0	50.6	5.0	27.8
Inadequate (< 2.4)	27	52.9	29.0	52.7	27.0	50.9	29.0	54.7	16.0	48.5	40.0	54.8	6.0	54.5	50.0	454.5	28.0	49.1	23.0	54.8	52.0	55.9		33.3	37.0	52.9	19.0	52.8	11.0	57.9	45.0	51.7	39.0	49.4	13.0	72.2

OR, odds ratio; CI, confidence interval; M, male; F, female; y, years; Alb, albumin; eGFR, estimated Glomerular filtration rate; Ca, calcium; P, phosphorus; K, potassium; UA, uric acid; hs-CRP, high sensitivity C-reactive protein; iPTH, intact parathyroid hormone; CHO, carbohydrate; SFAs, saturated fatty acids; PUFAs, polyunsaturated fatty acids; RE, retinal equivalent.

The analysis was adjusted for age, gender, post renal transplant vintage, glomerular filtration rate and Charlson comorbidity index. Significant level at $^{\dagger}P < 0.05$.

Nutrients of which the intake was inadequate: monounsaturated fatty acids, dietary fiber, calcium, magnesium, zinc, and folic acid

Blank: inadequate subjects to analysis.
